# First molecular characterization of *Echinococcus granulosus* (sensu stricto) genotype 1 among cattle in Sudan

**DOI:** 10.1186/s12917-018-1348-9

**Published:** 2018-02-01

**Authors:** Mohamed E. Ahmed, Bashir Salim, Martin P. Grobusch, Imadeldin E. Aradaib

**Affiliations:** 1 EBH Research Center, Zamzam University College (ZUC), Khartoum, Sudan; 2 Department of Parasitology, Faculty of Veterinary Medicine, University of Khartoum, Khartoum, Sudan; 3 Center of Tropical Medicine and Travel Medicine, Department of Infectious Diseases, Division of Internal Medicine, Amsterdam Medical Center, University of Amsterdam, Amsterdam, The Netherlands; 4 Molecular Biology Laboratory (MBL), Department of Clinical Medicine, Faculty of Veterinary Medicine, University of Khartoum, P.O. Box 32, Khartoum North, Sudan

## Abstract

**Background:**

*Echinococcus granulosus* sensu *lato* (*s.l.)* is the causative agent of cystic echinococcosis (CE), which is a cosmopolitan zoonotic parasitic disease infecting humans and a wide range of mammalian species including cattle. Currently, little information is available on the genetic diversity of *Echinococcus* species among livestock in Sudan. In the present study, fifty (*n* = 50) hydatid cysts were collected from cattle carcasses (one cyst sample per animal) at Al-kadarou slaughterhouse, Khartoum North, Sudan. DNA was extracted from protoscolices and the germinal layer of each cyst and subsequently amplified by PCR targeting the mitochondrial NADH dehydrogenase subunit 1 (NADH-1) gene. The amplified PCR products were purified and subjected to direct sequencing for subsequent construction of phylogenetic tree and net work analysis.

**Results:**

The phylogenetic tree revealed the presence of *Echinococcus canadenesis* genotype 6 (G6) in 44 cysts (88.0%), *Echinococcus ortleppi* genotype 5 (G5) in 4 cysts (8.0%) and *Echinococcus granulosus* sensu stricto (*s.s*) genotype 1 (G1) in 2 cysts (4.0%). The phylogenetic network analysis revealed genetic variation among the different haplotypes/genotypes. This report has provided, for the first time, an insight of the role of cattle in the transmission of the zoonotic G1 echinococosis.

**Conclusions:**

The results of the study illustrate that Sudanese breeds of cattle may play an important role in the transmission dynamics and the epidemiology of cystic echinococcosis in Sudan. This study reports the first molecular identification of *E. granulosus s.s.* in cattle in Central Sudan.

## Background

Cystic echinococcosis (CE) is a significant public health problem with high endemicity in east and central Africa including Sudan [1–4]. The larval stage of *Echinococcus granulosus* sensu *lato* (*s.l*.) causes CE in humans and a wide range of mammalian species. The life cycle involves the ingestion of parasite eggs by an intermediate host belonging to wildlife and domestic livestock species, including cattle. The dog is considered as the definitive host for this parasitic infection [5]. Humans are accidental dead end hosts. It is estimated that CE results in economic losses in the livestock sector due to morbidity. In addition, partial or total condemnations of infected organs of slaughtered animals are frequently encountered in endemic areas [5–9]. Echinococcosis has recently been included by the World Health Organization (WHO) as a neglected tropical disease [10]. CE may significantly affect the overall development and work productivity in endemic areas. In pastoral Sudanese communities, CE remains highly endemic with higher prevalence compared to agricultural communities. CE is endemic in most parts of the world, including regions of South America, the Mediterranean, Eastern Europe, East Africa, the Near and Middle East, Central Asia, China and Russia [7, 10–13]. Currently, ten distinct genotypes of *E. granulosus s.l.,* designated as G1-G10, have been described worldwide on the basis of genetic diversity related to nucleotide sequences of the mitochondrial NADH dehydrogenase subunit 1 (NADH 1) and cytochrome C oxidase subunit 1 (COX1) genes. These different genotypes are associated with distinct intermediate hosts including sheep, goats, horses, cattles, pigs, camels and and members of the cervid family [14–19]. Of the ten genotypes of *E. granulosus s.l*., the cattle (G5) and the camel (G6) strains have already been reported among humans and livestock in Sudan [2, 20, 21]. Recent epidemiological studies indicated that the camel genotype (G6) was the most prevalent strain in Sudan [4, 22]. The extensive intra-specific genetic variation of *E. granulosus s.l.* could be better understood within the context of variations in the life cycle pattern [23, 24]. It is suggested that, different genotypes would probably exhibit different antigenicity, transmission profiles, pathological consequences, and different sensitivity to chemotherapeutic agents [25]. A lot of research efforts have been directed towards the epidemiology of CE in Sudan [26–29]. However, only few reports of the genetic diversity of the parasite among the cattle in Sudan employed sequence analysis of mitochondrial markers [2, 4, 21]. It is, therefore, becoming increasingly obvious that expanding the existing sequence data on the genetic diversity of *E. granulosus s.l*. is necessary to better understand the biology, ecology and molecular epidemiology of this parasite. In this investigation, a molecular characterization was conducted to identify hydatid cysts recovered from local cattle breed in Central Sudan.

## Methods

### Collection of samples and processing

Fifty hydatid cysts (*n* = 50) were collected over a period of 6 months from cattle during April–October, 2016, at Al-kadarou slaughterhouse, Khartoum North, Central Sudan. This slaughter house is the major cattle battoir in Khartoum North, Sudan. Hydatid cysts were obtained from cattle instantly after slaughtering and transferred in thermo-flasks to the Molecular Biology Laboratory at the Faculty of Veterinary Medicine, University of Khartoum, for processing and molecular characterization.

### DNA extraction from hydatid cysts

Parasite genomic DNA was extracted from hydatid cysts as described by Ahmed and his coworkers [4]. Maximum DNA yield was obtained by spinning at 12,000 rpm for 1 min at room temperature. From the suspended nucleic acid, 5 μl was used in the PCR amplification.

### Primers design and PCR assays

The primers were designed based on the published sequences of NADH dehydrogenase subunit 1 (NADH-1) gene of *E. granulosus* genotype 6 (G6) reported by Bowles and McManus [15]. Briefly, primer EGL1: 5′TGA AGT TAG TAA TTA AGT TTA A′3 and primer EGR2: 5′AAT CAA ATG GAG TAC GAT TA′3 were designed to amplify a fragment of 435 bp of *E. granulosus s.l.* by PCR. The details of PCR amplification, visualization and of results were described previously [4].

### Sequence processing and phylogenetic analysis

The PCR products were purified using QIAquick PCR purification kit (Felden, Germany) and submitted for sequencing to a commercial company (Macrogen, Seoul, Korea). Bidirectional sequence fragments of the forward and reverse primers were generated for each sample. These were edited manually to correct possible base calling errors using BIOEDIT 7.0 and were subsequently joined to reconstruct a fragment of 344 bp of the parasite (NADH-1) gene. The consensus sequences were aligned with the corresponding region of NADH-1 gene of known genotypes circulating globally using CLUSTAL-X 2.1 [30]. The phylogenetic tree was constructed using the unweighted pair group method with arithmetic mean (UPGAM) implemented in MEGA software version 6.0 with 1000 bootstrap replicates [31]. Corresponding nucleotide sequences of NADH-1 of *Taenia multiceps* with GenBank accession number HM143887 were used as out groups in the constructed phylogenetic trees.

### Phylogenetic network analysis

To measure the genetic variability, the number of haplotypes was determined using DNASP v5 [31] with insertions and deletions considered as variable sites. We used the median-joining (MJ) network algorithm [32] implemented in NETWORK 4.6 (www.fluxus-engineering.com).

## Results and discussion

**Fig. 1:**
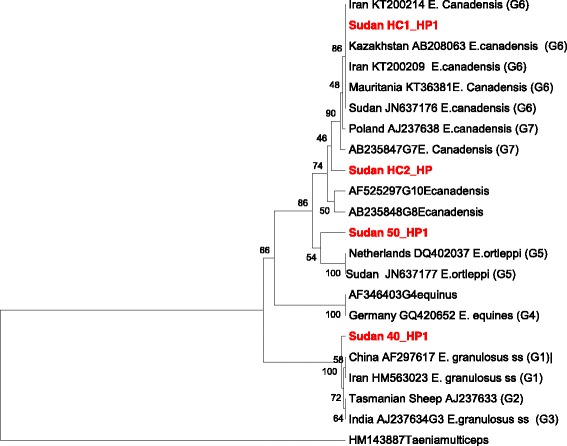
Phylogenetic relationship of hydatid cysts of *Echinococcus* granulosus sensu lato recovered from Sudanese cattle and other genotypes identified globally. NADH dehydrogenase subunit 1 (NADH-1) partial sequences generated from this study were aligned with sequences of other strains from different parts of the world. Sequences were analyzed with the BioEdit software (Ibis Biosciences, Carlsbad, CA, USA). The phylogenetic tree was constructed using unweighted pair group method with arithmetic mean (UPGMA) implemented in MEGA software version 6.0 [31]. Bootstrap values were calculated from analysis of 500 replicates of the data set, and values greater than 50% are indicated at the appropriate nodes. Each genotype was designated by its GenBank accession number and the country of origin when available. The GenBank accession numbers (LC167080, LC167081) were given for *Echinococcus granulossus* sensu stricto (G1) and *Echinococcus orteleppi* (G5), respectively. *Echinococcus canadensis* genotypes (G6) were given accession numbers LC167082 and LC167083). Corresponding nucleotide sequence of NADH 1 of *Taenia multiceps*, GenBank accession number HM143887, was used as an out group. The partial NADH-1gene sequences identified in this study were highlighted in red color for clarity of the constructed phylogenetic tree

**Fig. 2:**
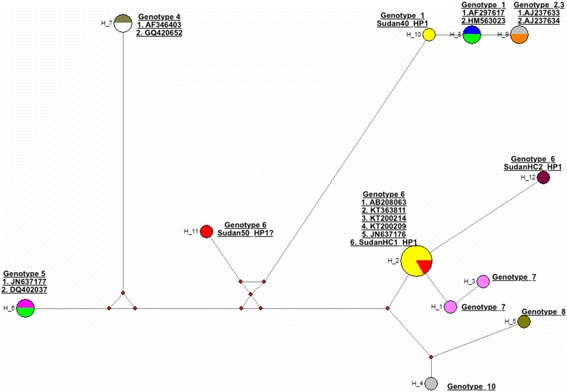
Phylogenetic network analysis of haplotypes. The number of haplotypes was determined with insertions and deletions considered as variable sites. Median-joining (MJ) network algorithm [32] implemented in NETWORK 4.6 was used to construct the phylogenetic network. The GenBank accession numbers were the same as indicated for the phylogenetic tree

Microscopic examination revealed that all hydatid cysts were fertile and measured 2–10 cm in diameter. The predilection sites of the cysts were found to be the lung and the liver. All fifty DNA samples were amplified by PCR and generated a fragment of 435 bp of the NADH-1 gene. The partial sequences of the NADH-1 gene representing genotypes G1 (accession number LC167080), G5 (accession number LC167081) and G6 (accession numbers LC167082 and LC167083) were submitted to GenBank, DNA Data Base of Japan (DDBJ). The sequence analysis indicated a prevalence of (88.0%, *n* = 44), (8.0%, n = 4), (4.0%, *n* = 2) for *Echinococcus canadenesis* (G6), *Echinococcus ortleppi* (G5), and *E.granulosus* sensu stricto s.s (G1), respectively. The phylogenetic network analysis revealed clear genetic variation between the different genotypes and haplotypes. The present investigation indicated that at least three different genotypes of *E. granulosus s.l.* are actively circulating in cattle in Sudan as illustrated by the phylogenetic tree (Fig. 1) and phylogenetic network analyses (Fig. 2). The sample Sudan HC1_HP was well grouped with haplotype 6 and samples SudanHC2_HP1 that was clustered with genotype 6 in the phylogenetic tree was six SNPs different from the haplotype 6. Sudan 40_HP was grouped with genotype 1 and differed with only 2 SNPs from the previously known genotype 1.The three *Echinococcus* genotypes (G1, G5 and G6) reported in this study are all known human pathogens of significant public health concern [33]. The exclusive occurrence and a predominant circulation of the camel genotype (G6) in the bovine species suggested that cattle can play an important role in the transmission dynamic and the epidemiology of the disease [4]. The present study indicated that *E. granulosus s.s.*, the sheep strain (G1), should equally be considered as an important infectious form of CE among cattle in Central Sudan.

## Conclusions

The present study represents the first molecular record of *E. granulosus s.s* G1, thus reinforcing its role as a source of infection among Sudanese cattle breeds. In addition, this investigation provides additional information on the existing data indicating that *Echinococcus granulosus s.s.* G1, which was previously restricted to other region in the African continent, is now becoming broadly distributed in the country. Active surveillance is required to determine the distribution and prevalence of CE and to identify the genotypes/strains circulating in different regions of Sudan.

## Abbreviations

CE: Cystic echinococcosis
cox1: Cytochrome C oxidase subunit 1
DDBJ: DNA Data Base of Japan
*E.granulosus s.l.*: *Echinococcus granulosus* sensu *lato*
*E.granulosus s.s.*: *Echinococcus granulosus* sensu *strict*: s.s
G1: Genotype 1
G5: Genotype 5
G6: Genotype 6
N: Number
NADH-1: NADH dehydrogenase subunit 1
